# Correlation Between Tumor-Associated Macrophage and Immune Checkpoint Molecule Expression and Its Prognostic Significance in Cutaneous Melanoma

**DOI:** 10.3390/jcm9082500

**Published:** 2020-08-03

**Authors:** Young Jae Kim, Chong Hyun Won, Mi Woo Lee, Jee Ho Choi, Sung Eun Chang, Woo Jin Lee

**Affiliations:** Department of Dermatology, Asan Medical Center, University of Ulsan College of Medicine, Seoul 05505, Korea; assayoungjae@naver.com (Y.J.K.); chwon98@chol.com (C.H.W.); miumiu@amc.seoul.kr (M.W.L.); jhchoy@amc.seoul.kr (J.H.C.)

**Keywords:** melanoma, programmed cell death protein-1, lymphocyte activating gene-3, CD163, prognosis, immunotherapy

## Abstract

The association between tumor-associated macrophages (TAMs) and the expression of immune checkpoint molecules has not been well described in cutaneous melanoma. We evaluated the correlations between the expression of markers of TAMs, cluster of differentiation 163 (CD163), and immune checkpoint molecules, programmed cell death protein-1 (PD-1), and lymphocyte activating gene-3 (LAG-3). We also determined their relationships with the clinicopathological features and disease outcomes in melanoma. Diagnostic tissues collected from melanoma patients were evaluated using immunohistochemistry for CD163, PD-1, and LAG-3 expression. CD163 expression positively correlated with PD-1 and LAG-3 expression. High expression of both CD163 and PD-1 expressions was significantly associated with negative prognostic factors and worse prognosis than high expression of the single markers. High co-expression of CD163 and LAG-3 was associated with poor clinicopathological indexes of melanoma and worse survival compared to the high expression of the single markers. The expression of immune checkpoint molecules PD-1 and LAG-3 positively correlated with the M2-TAM density in melanoma tissue. Simultaneous high M2-TAM density and immune checkpoint molecules expression acted as independent poor prognostic factors in cutaneous melanoma.

## 1. Introduction

Programmed cell death protein-1 (PD-1) is a major inhibitory receptor expressed on T, B, and natural killer (NK) cells and mediating the suppression of antitumor immunity [1]. In the solid tumors microenvironment (TME), PD-1 expressed cells are increased, resulting in further tumor invasion and unfavorable prognosis [1,2,3]. Therefore, programmed cell death protein-1/ligand-1 (PD-1/PD-L1) signaling inhibitors act as representative immunotherapeutic agents that activate anti-tumor immune responses in various tumors [4]. Another emerging immune checkpoint molecule, lymphocyte activating gene-3 (LAG-3), revealed a synergistic immunosuppressive action with PD-1 [5].

Importantly, tumor-associated macrophages (TAMs) have recently attracted considerable attention since macrophages comprise the predominant population of tumor-infiltrating inflammatory cells [2]. Two main types of macrophages have been reported, pro-inflammatory (M1) or anti-inflammatory (M2) macrophages [2,6]. These two types of macrophage can be distinguished using HLA-DR and cluster of differentiation 163 (CD163 staining, respectively [7]. In particular, CD163-positive M2 macrophages play a role in promoting tumor development by suppressing antitumor immunity [8]. Representative immune cells, such as dendritic cells, neutrophils, and regulatory T cells interact with CD163-positive M2 macrophages [9].

Although the prognostic value of CD163 and PD-1 expression in cutaneous melanoma has been reported, the association between the expression of CD163 and PD-1 in melanoma tissue remains unclear [8]. Colon cancer TAMs from both mouse model and human specimens express PD-1, and high levels of PD-1 have been shown to inhibit phagocytosis and tumor immunity [10]. A recent study in lung adenocarcinoma also showed similar expression pattern of PD-1 by TAMs in addition to PD-1 expression on T cells in both mouse cells and human tissues [11]. In the context of melanoma, it was reported that CD163-positive TAMs can suppress antitumor immunity in anti-PD-1-resistant melanoma [8].

The prognostic significance of LAG-3 in cutaneous melanoma was reported. [12] CD163 positive M2-TAMs have been shown to induce the expression of LAG-3, PD-1, and TIM-3 on CD8+ T cells in vitro [13,14]. However, also the relationship between the expression of CD163 and LAG-3 in cutaneous melanoma has not yet been investigated.

In this study, we investigated the correlation between the density of M2-TAMs and the immune checkpoint molecules PD-1 and LAG-3 by using immunohistochemistry on melanoma tissue. We also determined their relationships with the clinicopathological features and disease outcomes in melanoma.

## 2. Experimental Section

### 2.1. Study Design

The study protocol was approved by our Institutional Review Board (2017-012). The Asan Medical Center database was investigated for cases of cutaneous melanoma that were confirmed by skin biopsy between January 2002 and June 2016. Cases of in situ melanoma that were unsuitable for microscopic evaluation were excluded.

### 2.2. Histopathological Analysis and Immunohistochemistry

All the histological and immunophenotypic data pertaining to the 102 cases were reviewed. The biopsy slides were reviewed, and the following parameters were analyzed: Breslow thickness, ulceration, and vertical growth phase.

Formalin-fixed, paraffin-embedded tumor samples were incubated with antibodies against cleaved CD163 (marker of M2-TAMs) (dilution 1:400;NOVO, Newcastle, UK), PD-1 (1:100, Ventana, Tucson, AZ, USA), and LAG-3 (1:2000, Abcam, Cambridge, UK). We examined the protein expression levels of CD163, PD-1, and LAG-3 in 5% increment cases. The relative percent of CD163, PD-1 or LAG-3 positive cells within to the overall tumor infiltrating inflammatory cells was semi-quantitatively calculated. Melan-A stain was used to identify melanoma cells. The two dermatologists (YJK, WJL) agreed on LAG-3 expression in 90 of 102 (88%, k = 0.653) tumors, on CD163 expression in 90 of the 102 (88%, k = 0.653) and on PD-1 expression in 91 (89%, k = 0.639) tumor. Samples with consensus by both the investigators have been included. The cutoff values for high expression were selected based on values with the maximum significant differences in overall survival (OS) or values from a previous study [12]. A sample was considered to show high expression for CD163 if the positive cells constituted ≥35% of the overall cellularity. A sample was considered to show high PD-1 and LAG-3 expression if ≥20% of the tumor infiltrating inflammatory cells demonstrated reactivity to the anti-PD-1 and LAG-3 antibody.

### 2.3. Statistical Analyses

OS was calculated based on the date of initial diagnosis and either the date of death or the date of the last follow-up examination. Progression-free survival (PFS) was calculated based on the date of initial diagnosis and the date of the first day of disease progression, disease recurrence, or the last follow-up examination. Survival curves were generated based on the medical records. The disease stage was determined according to the American Joint Committee on Cancer (AJCC) classification [15]. The clinicopathological features and disease outcomes were estimated according to the different expression level of CD163, PD-1 and LAG-3.

### 2.4. Variables of Interest

Survival analyses were performed using the Kaplan–Meier method with the log-rank test. Prognostic factors that were independently associated with the OS were identified using multivariate analysis with a Cox proportional-hazards regression model. Subgroup comparisons were performed using either chi-squared test (categorical variables) or *t*-test (continuous variables). Pearson’s correlations were used to investigate the associations among the continuous variables. For each dataset, the cutoff point that best segregated patients into poor and good prognosis subgroups was determined using a maximum chi-squared test. All the analyses were performed using the SPSS statistical package (version 18.0; SPSS Inc., Chicago, IL, USA). A *p*-value of <0.05 was considered statistically significant.

## 3. Results

A total of 102 cases of cutaneous melanoma were included in the study. The demographic data and clinical features of the patients are summarized in Table 1.

### 3.1. Correlation between CD163, PD-1, and LAG-3 Expression in Melanoma Tissue

**Figure 1:**
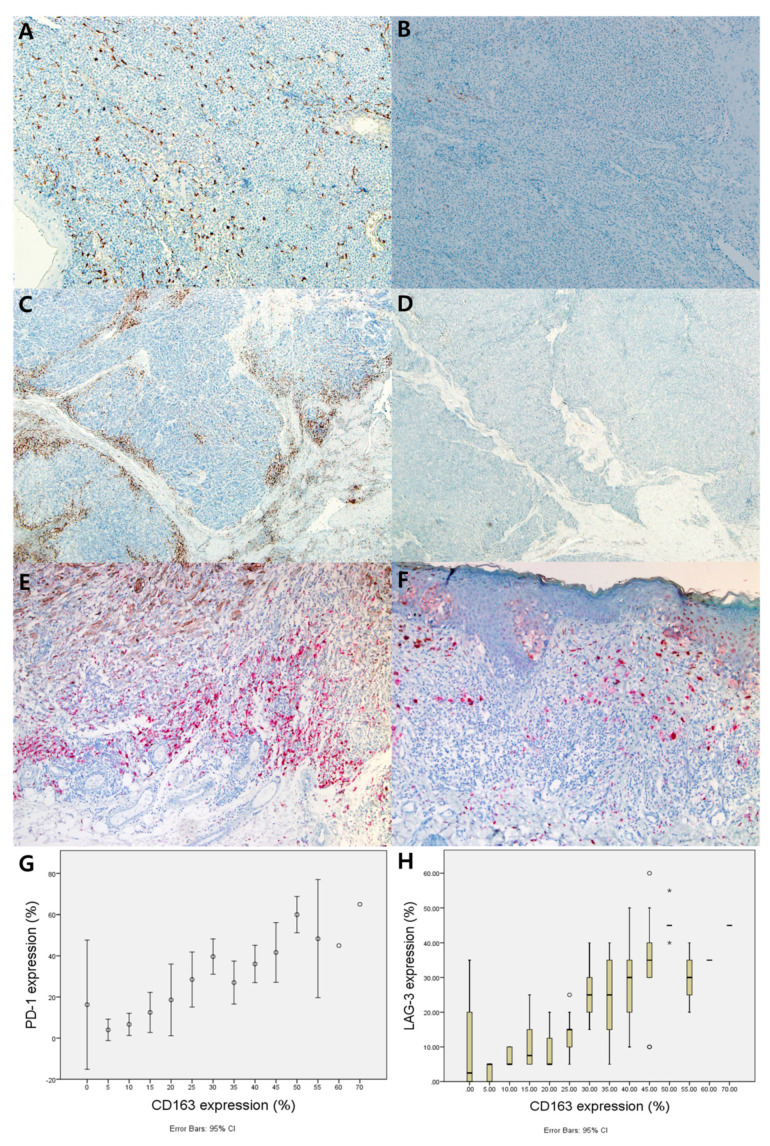
High expression for CD163 and PD-1, LAG-3 and their correlation. (**A**) Representative sections of high expression for CD163. (**B**) Low expression for CD163. (**C**) Representative sections of high expression for PD-1. (**D**) Low expression for PD-1. (**E**) Representative sections of high expression for LAG-3. (**F**) Low expression for LAG-3. (**G**) Correlation between CD163 and PD-1 in cutaneous melanoma; (rho 0.621, *p* < 0.001) Pearson’s correlations were used to investigate the associations among the continuous variables. (**H**) Correlation between CD163 and LAG-3 in cutaneous melanoma; (rho 0.702, *p* < 0.001) Pearson’s correlations were used to investigate the associations among the continuous variables.

Of 102 patients, CD163 and PD-1 were highly expressed in 47 (46.1%) and 53 (52.0%) patients, respectively (Figure 1 A,C). High expression of LAG-3 was found in 44 (43.1%) patients (Figure 1E). Patients with high expression of both CD163 and PD-1 were 32 (31.4%) patients. Concurrent high expression of both CD163 and LAG-3 was found in 32 (31.4%) patients. Among the 53 patients with a high expression of PD-1, 32 (60.4%) patients revealed a high expression of CD163. Of the 44 patients with a high expression for LAG-3, 32 (72.7%) patients revealed a high expression of CD163. There was a significant association between CD163 and both PD-1 expression (*p* = 0.003) and LAG-3 expression (*p* < 0.001) (Table 2). When these expression values were measured as continuous variables, CD163 expression exhibited a positive correlation with both PD-1 (Figure 1G) and LAG-3 (Figure 1H) (PD-1; rho 0.621, *p* < 0.001, LAG-3; rho 0.702, *p* < 0.001).

### 3.2. Expression of CD163, PD-1, LAG-3, and Their Association With Clinicopathological Features

Clinicopathological variables were stratified base on the tumor expression of CD163, PD-1, and LAG-3 (Table 1). High CD163 expression was associated with poor clinicopathological variables, such as a deeper Breslow thickness (*p* = 0.001), frequent vertical growth phase (*p* = 0.001), higher likelihood of lymph node(LN) involvement (*p* = 0.005), and an advanced AJCC stage (*p* = 0.012; Table 1).

Concurrent high expression of both CD163 and PD-1 (CD163^high^PD-1^high^) was associated with clinicopathological variables that have negative prognostic indexes, such as a deeper Breslow thickness (*p* = 0.001) and higher frequency of vertical growth phase (*p* < 0.001). High expression of both CD163 and PD-1 expression was also associated with a higher likelihood of LN involvement (*p* = 0.033), visceral involvement (*p* = 0.048), and an advanced AJCC stage (*p* = 0.028; Table 1).

As for PD-1, there was a significant association between simultaneous high expression of both CD163 and LAG-3(CD163^high^LAG-3^high^) and poor clinicopathological variables, including a deeper Breslow thickness (*p* = 0.011) and higher frequency of vertical growth phase (*p* < 0.001), a higher likelihood of LN involvement (*p* = 0.008), and an advanced AJCC stage (*p* = 0.007; Table 1).

### 3.3. Prognostic Significance of CD163, PD-1, and LAG-3 Expression

**Figure 2:**
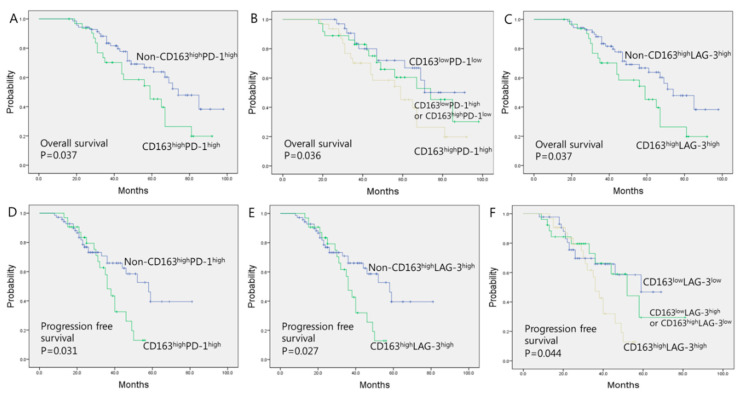
Patient survival outcome according to expression of CD163, PD-1, and LAG-3 in cutaneous melanoma. (**A**) OS according to high expression of both CD163 and PD-1. (Hazard ratio (HR); 2.08, 95%CI; 1.14-5.57). (**B**) Worse OS in patients with high expression of both CD163 and PD-1. (**C**) OS according to high expression of both CD163 and LAG-3. (HR; 1.88, 95%CI; 1.16–5.87). (**D**) PFS according to high expression of both CD163 and PD-1. (HR; 1.76, 95%CI; 1.08–5.46). (**E**) PFS according to high expression of both CD163 and LAG-3. (HR; 1.54, 95%CI; 1.07–5.13). (**F**) Worse PFS in patients with high expression of both CD163 and LAG-3. A sample was considered to show high expression for CD163 if the positive cells constituted ≥35% of the overall cellularity. A sample was considered to show high PD-1 and LAG-3 expression if ≥20% of the tumor infiltrating inflammatory cells. Survival analyses were performed using the Kaplan–Meier method with the log-rank test. Multivariate analyses were performed using a Cox proportional-hazards regression model.

The follow-up period of the patients ranged from 22–149 months (median follow-up: 76 months). The 5-year OS rate was 48%, and the mean OS was 66.9 months [95% confidence interval (CI), 60.67–73.05 months]. The survival outcomes were significantly worse when CD163 and tumor immune checkpoint molecules was concurrently high. The mean OS was significantly poorer in patients with high expression of both CD163 and PD-1 (CD163^high^PD-1^high^) (57.57 months; 95% CI, 48.49–66.66 months) compared with non-CD163^high^PD-1^high^ (71.16 months; 95% CI, 63.49–78.82 months) (*p* = 0.037, Figure 2A) (Table 3). Patients with either high expression of CD163 or PD-1 showed inferior mean OS (68.17 months; 95% CI, 57.36–78.98 months) than those with low expression of both CD163 and PD-1 (CD163 ^low^PD-1^low^) (70.94 months; 95% CI, 61.78–80.10 months) (*p* = 0.036, Figure 2B). OS was significantly worse when the expression of CD163 and LAG-3 concurrently high (CD163^high^LAG-3^high^) (OS: *p* = 0.037, Figure 2C) (Table 3). Patients with either high expression of CD163 or LAG-3 revealed inferior mean OS (70.35 months; 95% CI, 57.80–82.89 months) than those with low expression of both CD163 and LAG-3 (CD163^low^LAG-3^low^) with a marginal statistical significance (68.53 months; 95% CI, 60.20–76.85 months) (*p* = 0.064). PFS was also significantly worse in patients with high expression of both CD163 and PD-1 (*p* = 0.031, Figure 2D). PFS was also affected by the expression of either CD163 or PD-1 (*p* = 0.031). PFS was significantly worse when the expression of CD163 and LAG-3 concurrently high (CD163^high^LAG-3^high^) (PFS: *p* = 0.027, Table 3, Figure 2E). PFS was inferior in patients with either high expression of CD163 or LAG-3 than those with low expression of both CD163 and LAG-3 (CD163^low^LAG-3^low^) (*p* = 0.044, Figure 2F).

Univariate analysis revealed that high expression of both CD163 and PD-1 (CD163^high^PD-1^high^) expression (hazard ratio (HR) = 2.31, 95% confidence interval (CI) = 1.21–5.11, *p* = 0.029) was associated with a lower OS (Table 4). Moreover, the simultaneous high expression of both CD163 and LAG-3 (CD163^high^LAG-3^high^) affected OS (hazard ratio (HR) = 2.03, 95% confidence interval (CI) = 1.18–5.44, *p* = 0.032). Multivariate analysis revealed that high expression of both CD163 and PD-1 (CD163^high^PD-1^high^) expression (HR = 2.08, 95% CI = 1.14–5.57, *p* = 0.042) and for both CD163 and LAG-3 (CD163^high^LAG-3^high^) expression (HR = 1.88, 95% CI = 1.16–5.87, *p* = 0.044) were independent prognostic markers for lower OS.

## 4. Discussion

CD163 is a scavenger receptor for haptoglobin-hemoglobin complexes [16] specifically expressed on M2 macrophages. This marker has been investigated as an indicator of poor prognosis in various tumors, including melanoma [16], breast [17], gastric [18], ovarian [19], pancreatic cancer [20], as well as head and neck squamous carcinoma [21]. Recent studies in advanced cutaneous melanoma also revealed that increased serum levels of CD163 were related to positive response to anti-PD-1 antibody [22].

As a prognostic marker in various cancers, understanding the role of CD163-positive TAMs in TME is essential. First, CD163-positive TAMs could affect the tumor-infiltrating lymphocytes (TILs) in TME. When M2-TAMs was depleted, there was a marked increase in the numbers of CD4+ and CD8+ TILs in melanomas [8]. In colorectal cancers, high expression of CD163 on TAMs was found in TME, resulting in an increased number of CD4+ lymphocytes that contributed to up-regulate the PD-1 expression [23]. Moreover, M2-TAMs produced tumor-promoting cytokines, such as interleukin (IL)-6 and IL-10, which suppress cytotoxic TIL [8,23]. These tumor prone macrophages could express the transcription factor IRF4 [23], which promoted CD8+ T cell exhaustion and acts as an up-regulator of PD-1 expression [24]. Exhausted CD8+ T cells within the TME showed increased expression of inhibitory receptors, particularly PD-1, LAG-3, T-cell immunoglobulin, and mucin-domain containing 3 (TIM-3) [14]. Noy et al. reported that M2-TAMs could suppress CD8+ T cells through directly interacting with T cells by the PD-1/PD-L1 signaling or by secreting immunosuppressive cytokines, such as TGF-β and IL-10. [25] In a study focused on melanoma, CD163-positive M2-TAMs could block the recruitment of antitumor CD8+ T cells [8]. In sum, CD163-positive M2-TAMs may suppress the cytotoxic activity and inhibit the recruitment of CD8+ T cells, enabling tumor escape from antitumor immunity.

In our previous study, high CD163 expression was associated with lower OS, suggesting the prognostic significance of CD163 expression in cutaneous melanoma [16]. We observed that CD163 expression correlated with VEGF and COX-2 expression [16]. However, limited information is available about the expression of CD163 and its relationship with immune checkpoint molecules, particularly PD-1 and LAG-3. In this study, 46.1% of melanoma specimens showed high expression of CD163. In addition, high expression of CD163 was significantly associated with both PD-1 and LAG-3 expression. This suggests that PD-1 and LAG-3 expression could be affected by CD163-positive TAMs in cutaneous melanoma. Consistent with our findings, PD-1 and CD163 correlation as checkpoint markers was found in renal cell carcinoma [6]. In addition, LAG-3 expression closely correlated with CD163 in primary head and neck squamous cell carcinoma [26].

In the present study, PD-1 expression itself did not show any association with poor prognosis (data are not shown). The same result was consistently found in our previous study dealing with the prognostic significance of PD-1 expression in cutaneous melanoma [27]. In the present study, we found two notable results; first, PD-1 and LAG-3 correlated with the CD163 expression, suggested as a poor prognostic factor in melanoma. Second, the concurrent high expression of both PD-1 and CD163 (CD163^high^PD-1^high^) correlated worst survival outcomes. The concurrent high expression of both LAG-3 and CD163 (CD163^high^LAG-3^high^) also revealed an association with worst survival outcomes. PD-1 can be expressed both in activated and exhausted T cells; therefore, it is difficult to evaluate the genuine antitumor exhausted immune status provoked in tumor cells when it is investigated only considering PD-1 expression [12]. As described, CD163 positive M2-TAMs can induce the expression of PD-1, LAG-3, and TIM-3 in CD8+ T cells [14]. In melanoma patients, PD-1+/TIM-3+ status could indicate an exhausted T-cell phenotype [28]. Therefore, we believe that high CD163 expression identifies the activation of M2-TAMs on TME, possibly distinguishing the expression of PD-1 in exhausted T cells.

However, one should be cautious in concluding that CD163 and PD-1 expressions were associated in an independent manner. It was reported that TAMs by itself can express high levels of PD-1 in colon cancer, and that PD-1 expression augments with the disease stage [10]. PD-1 expressed on macrophages decreased the level of the IL-6 [29] and IL-12 [30] cytokine secretion, which induced M2 polarization. Moreover, anti-PD-1 therapy may repolarize macrophages from M2 to M1 phenotype [31]. This means that PD-1/PD-L1 signaling is closely associated with macrophage reprogramming process in TME. According to this, increased PD-1 expression correlates with decreased phagocytosis, resulting in an increased tumor burden for colorectal cancer [10]. In sum, we believe that PD-1 and CD163 were positively correlated and worked in a synergistic manner.

Patients who were non-responsive to anti-PD-1 therapies often showed a correlation with low TIL recruitment in primary tumors [32]. M2-phase polarization of macrophages is associated with the suppression of TIL function [33]; immunotherapies targeting the redirection of macrophage polarization from M2 into M1 could be theoretically plausible. In our study, with respect to high PD-1 expression, patients with high expression of CD163 (CD163^high^PD-1^high^) had a poorer prognosis than those with low expression of CD163 (CD163^low^PD-1^high^). Based on these findings, CD163 expression can stratify survival in patients with high expression of PD-1.

In regard to LAG-3 expression, the same results were observed according to high CD163 expression as well. In our previous study, LAG-3 expression correlated with PD-1 expression in melanoma, potentially acting as a prognostic biomarker [12]. LAG-3 can regulate T cell proliferation, activation, and homeostasis, leading to consider it as a promising therapeutic candidate for immune therapy in various cancers [34]. Our findings support the notion that additive combination strategies with anti-macrophage immunotherapy may be effective in advanced melanoma.

In fact, similar combinatory strategies that target M2-macrophages have been reported. Macrophage receptor with collagenous structure (MARCO), a pattern-recognition receptor of the class A scavenger receptor family, was suggested as an immunologic target. This MARCO expression correlated with the expression of M2-macrophage marker CD163. Anti-MARCO immunotherapy could synergistically enhance the effect of checkpoint inhibitor (anti-CTLA4) in B16 melanoma models [35]. However, as for human context, there was no clinical study dealing with M2-macrophage antibodies. Only one phase I/II clinical study has been investigated administrating anti-CTLA4 antibody with anti-CD40 agonist (CD40; M1-TAMs marker) in metastatic melanoma [2]. (NCT01103635)

## 5. Conclusions

In conclusion, the expression of PD-1 and LAG-3 correlated with the density of M2-TAMs and CD163 expression can stratify patients with high expression of PD-1 or LAG-3. Concurrent high expression of both CD163 and PD-1 (CD163^high^PD-1^high^) or LAG-3 (CD^163high^LAG-3^high^) in melanoma were independent poor prognostic factors. Our results may support combinatory immunotherapeutic strategies, especially for targeting anti-PD-1 and/or anti-LAG-3 with anti-CD163 or macrophage inhibitors in advanced cutaneous melanoma.

## Figures and Tables

**Table 1:** Clinical-histopathological characteristics of the 102 cutaneous melanomas related to CD163, PD-1, and LAG-3 expression.

	Total	CD163^High^			CD163^High^ PD-1^High^			CD163^High^ LAG-3^High^		
	*n* = 102	No (*n* = 55)	Yes (*n* = 47)	*p*-value	No (*n* = 70)	Yes (*n* = 32)	*p*-value	No (*n* = 70)	Yes (*n* = 32)	*p*-value
**Age (years)**				0.455			0.662			0.624
**Range**	25–89	25–80	31–89		25–89	31–86		25–89	31–82	
**Mean**	61.8	60.8	62.1		61.4	62.0		61.3	62.4	
**Sex**				0.769			0.705			0.419
**Male**	57	30 (54.5)	27 (57.4)		40 (57.1)	17 (53.1)		41 (58.6)	16 (50.0)	
**Female**	45	25 (45.5)	20 (42.6)		30 (42.9)	15 (46.9)		29 (41.4)	16 (50.0)	
**Breslow thickness, mm**				0.001 *			0.001 *			0.011 *
≤1 (T1)		15/55 (27.2)	6/47 (12.8)		T1 or T2	T1 or T2		T1 or T2	T1 or T2	
>1 to ≤2 (T2)		24/55 (43.6)	12/47 (25.5)		47/70 (67.1)	10/32 (31.3)		45/70 (64.3)	12/32 (37.5)	
>2 to ≤4 (T3)		10/55 (18.2)	16/47 (34.0)		T3 or T4	T3 or T4		T3 or T4	T3 or T4	
>4 (T4)		6/55 (10.9)	13/47 (27.7)		23/70 (32.9)	22/32 (68.8)		25/70 (35.7)	20/32 (62.5)	
**Ulceration**				0.591			0.659			0.173
No	70	39/55 (70.9)	31/47 (66.0)		49/70 (70.0)	21/32 (65.6)		51/70 (72.9)	19/32 (59.4)	
Yes	32	16/55 (29.1)	16/47 (34.0)		21/70 (30.0)	11/32 (34.4)		19/70 (27.1)	13/32 (40.6)	
**Vertical growth phase**				0.001 *			<0.001 *			<0.001 *
No	61	41/55 (74.5)	20/47 (42.6)		51/70 (72.9)	10/32 (31.3)		50/70 (71.4)	11/32 (34.4)	
Yes	41	14/55 (25.5)	27/47 (57.4)		19/70 (27.1)	22/32 (68.8)		20/70 (28.6)	21/32 (65.6)	
**Lymph node involvement**				0.005 *			0.033 *			0.008 *
No	80	49/55 (89.1)	31/47 (66.0)		59/70 (84.3)	21/32 (65.6)		60/70 (85.7)	20/32 (62.5)	
Yes	22	6/55 (10.9)	16/47 (34.0)		11/70 (15.7)	11/32 (34.4)		10/70 (14.3)	12/32 (37.5)	
**Visceral involvement**				0.087			0.048 *			0.237
No	94	53/55 (96.4)	41/47 (87.2)		67/70 (95.7)	27/32 (84.4)		66/70 (94.3)	28/32 (87.5)	
Yes	8	2/55 (3.6)	6/47 (12.8)		3/70 (4.3)	5/32 (15.6)		4/70 (5.7)	4/32 (12.5)	
**AJCC stage**				0.012 *			0.028 *			0.007 *
I/II	75	46/55 (83.6)	29/47 (61.7)		56/70 (80.0)	19/32 (59.4)		57/70 (81.4)	18/32 (56.3)	
III/IV	27	9/55 (16.4)	18/47 (38.3)		14/70 (20.0)	13/32 (40.6)		13/70 (18.6)	14/32 (43.8)	

* Statistically significant; PD-1, programmed cell death protein-1; LAG-3, lymphocyte activating gene-3; AJCC, American Joint Committee on Cancer. Subgroup comparisons were performed using either chi-squared test (categorical variables) or *t*-test (continuous variables).

**Table 2:** Correlations between CD163 and PD-1 or LAG-3 expression.

	PD-1 Expression, *n* (%)			LAG-3 Expression, *n* (%)		
	Low Expression (*n* = 49)	High Expression (*n* = 53)	*p*-Value	Low Expression (*n* = 58)	High Expression (*n* = 44)	*p*-Value
**CD163 expression**			0.003 *			<0.001 *
Low expression (*n* = 55)	34/49 (69.4)	21/53 (39.6)		43/58 (74.1)	12/44 (27.3)	
High expression (*n* = 47)	15/49 (30.6)	32/53 (60.4)		15/58 (25.9)	32/44 (72.7)	

* Statistically significant; PD-1, programmed cell death protein-1; LAG-3, lymphocyte activating gene-3. Comparisons were performed using chi-squared test.

**Table 3:** Survival outcomes of 102 patients with cutaneous melanoma according to CD163, PD-1, and LAG-3 expression.

	Mean OS (95% CI) (Months)	Mean PFS (95% CI) (Months)
A) CD163^low^PD-1^low^ expression (*n* = 34)	70.94 (61.78–80.10)	51.48 (43.41–59.55)
B) Either CD163^low^PD-1^high^ or CD163^high^PD-1^low^ expression (*n* = 36)	68.17 (57.36–78.98)	50.46 (39.54–61.39)
C) CD163^high^PD-1^high^ expression (*n* = 32)	57.57 (48.49–66.66)	37.06 (32.24–41.87)
D) CD163^low^LAG-3^low^ expression (*n* = 44)	68.53 (60.20–76.85)	50.19 (42.83–57.57)
E) Either CD163^low^LAG-3^high^ or CD163^high^LAG-3^low^ expression (*n* = 26)	70.35 (57.80–82.89)	50.93 (38.74–63.12)
F) CD163^high^LAG-3^high^ expression (*n* = 32)	57.57 (48.49–66.66)	36.89 (32.06–41.72)
**Overall** (*n* = 102)	66.86 (60.67–73.05)	48.39 (42.26–54.53)
***p*-value**		
A, B, C separately	0.036 *^,§^	0.026 *^,§^
C versus. others	0.037 *^,§§^	0.031 *^,§§^
D, E, F separately	0.064 ^ǂ^	0.044 *^,^^ǂ^
F versus. others	0.037 *^,^^ǂǂ^	0.027 *^,^^ǂǂ^

* Statistically significant. § *p*-value of the analysis for each three group of A, B, and C. §§ *p*-value of the analysis for two comparative groups between C and others. ǂ *p*-value of the analysis for each three group of D, E, and F. ǂǂ *p*-value of the analysis for two comparative groups between F and others. PD-1, programmed cell death protein-1; LAG-3, lymphocyte activating gene-3; CI, confidence interval; OS, overall survival; PFS, progression free survival.

**Table 4:** Univariate and multivariate analyses for overall survival and progression-free survival.

	Univariate analysis					
	OS			PFS		
Covariate	HR	95% CI	*p* Value	HR	95% CI	*p* Value
**Age (years)**						
<60 vs. ≥60	0.92	0.68–2.93	0.328	1.10	0.68–1.78	0.624
**Sex**						
Female vs. Male	1.84	0.67–3.28	0.412	1.28	0.79–4.57	0.492
**AJCC stage**						
Early vs. Advanced	2.41	1.24–4.28	0.023 *	2.19	1.14–5.37	0.045 *
**Involvement of LN**						
Yes vs. No	1.69	1.10–5.63	0.037 *	1.13	1.16–5.74	0.041 *
**Amelanosis**						
Yes vs. No	0.69	0.23–4.69	0.333	0.71	0.29–5.19	0.572
**Breslow thickness**						
T1	0.75	0.35–0.82	0.027 *	0.52	0.31–0.81	0.031 *
T2	0.82	0.05–1.18	0.141	0.86	0.23–1.11	0.062
T3	1.88	1.16–5.23	0.029 *	1.52	1.14–6.32	0.041 *
T4	2.41	1.11-4.82	0.018 *	1.99	1.19–5.81	0.039 *
**CD163 expression**						
Yes vs. No	2.62	1.39–4.88	0.033*	2.71	1.13–4.16	0.159
**LAG-3 expression**						
High vs. Low	2.11	1.21–5.20	0.037*	2.07	1.13–5.31	0.041 *
**PD-1 expression**						
High vs. Low	1.09	0.59–4.75	0.421	1.13	0.85–4.92	0.280
CD163^high^PD-1^high^	2.31	1.21–5.11	0.029*	1.88	1.07–5.09	0.043 *
CD163^high^LAG-3^high^	2.03	1.18–5.44	0.032 *	2.08	1.14–5.25	0.038 *
	**Multivariate analysis**					
**AJCC stage**						
Early vs. Advanced	1.89	1.21–4.59	0.042 *	1.87	1.11–4.21	0.051
**Involvement of LN**						
Yes vs. No	1.12	0.82–4.19	0.156	1.17	0.65–3.84	0.147
**Breslow thickness**						
T1	0.88	0.43–0.91	0.043 *	0.92	0.55–0.98	0.047 *
T2	0.89	0.27–1.47	0.117	0.84	0.33–1.61	0.215
T3	1.16	1.08–5.14	0.088	1.21	1.03–4.22	0.174
T4	1.89		0.041 *	1.61	1.10-4.87	0.063
**CD163 expression**						
High vs. Low	2.38	1.16–4.74	0.040 *	2.19	1.06–5.08	0.188
**LAG-3 expression**						
High vs. Low	1.99	1.19–5.44	0.044 *	1.87	1.10–5.01	0.061
**CD163^high^PD-1^high^**	2.08	1.14–5.57	0.042 *	1.76	1.08–5.46	0.066
**CD163^high^LAG-3^high^**	1.88	1.16–5.87	0.044 *	1.54	1.07–5.13	0.046 *

PD-1, programmed cell death protein-1; LAG-3, lymphocyte activating gene-3; AJCC, American Joint Committee on Cancer; LN, lymph node; CI, confidence interval; OS, overall survival; PFS, progression-free survival; * Statistically significant.
